# The ubiquitin ligase Cullin-1 associates with chromatin and regulates transcription of specific c-MYC target genes

**DOI:** 10.1038/s41598-020-70610-0

**Published:** 2020-08-18

**Authors:** Melanie A. Sweeney, Polina Iakova, Laure Maneix, Fu-Yuan Shih, Hannah E. Cho, Ergun Sahin, Andre Catic

**Affiliations:** 1 Department of Molecular and Cellular Biology, Baylor College of Medicine, Houston, TX USA; 2 Huffington Center on Aging, Baylor College of Medicine, Houston, TX USA; 3 Stem Cells and Regenerative Medicine Center, Baylor College of Medicine, Houston, TX USA; 4 Dan L. Duncan Comprehensive Cancer Center, Baylor College of Medicine, Houston, TX USA; 5 Center for Cell and Gene Therapy, Baylor College of Medicine, Houston, TX USA; 6 Rice University Undergraduate School of Social Sciences, Houston, TX USA; 7 Michael E. DeBakey Veterans Affairs Medical Center, Houston, TX USA

**Keywords:** Ubiquitin ligases, Gene expression

## Abstract

Transcription is regulated through a dynamic interplay of DNA-associated proteins, and the composition of gene-regulatory complexes is subject to continuous adjustments. Protein alterations include post-translational modifications and elimination of individual polypeptides. Spatially and temporally controlled protein removal is, therefore, essential for gene regulation and accounts for the short half-life of many transcription factors. The ubiquitin–proteasome system is responsible for site- and target-specific ubiquitination and protein degradation. Specificity of ubiquitination is conferred by ubiquitin ligases. Cullin-RING complexes, the largest family of ligases, require multi-unit assembly around one of seven cullin proteins. To investigate the direct role of cullins in ubiquitination of DNA-bound proteins and in gene regulation, we analyzed their subcellular locations and DNA-affinities. We found CUL4A and CUL7 to be largely excluded from the nucleus, whereas CUL4B was primarily nuclear. CUL1,2,3, and 5 showed mixed cytosolic and nuclear expression. When analyzing chromatin affinity of individual cullins, we discovered that CUL1 preferentially associated with active promoter sequences and co-localized with 23% of all DNA-associated protein degradation sites. CUL1 co-distributed with c-MYC and specifically repressed nuclear-encoded mitochondrial and splicing-associated genes. These studies underscore the relevance of spatial control in chromatin-associated protein ubiquitination and define a novel role for CUL1 in gene repression.

## Introduction

Mammalian gene expression follows oscillatory patterns, caused by alternating binding of transcriptional activators and repressors to specific DNA elements^1^. The exchange of regulators during such cycles is partially accomplished through protein removal and subsequent degradation by the ubiquitin–proteasome system^2^. Therefore, it is not surprising that transcription factors and their co-regulators are among the most short-lived proteins^3^. However, the specific factors that trigger the removal of chromatin-associated proteins, and the genomic locations of degradation remain ill-defined^4^.

The ubiquitin–proteasome system is a multi-enzyme cascade that triggers the covalent attachment of ubiquitin polypeptides to target proteins. Ubiquitination can impact protein function and trafficking, or mark proteins for proteasomal digestion. The ubiquitin–proteasome system is responsible for the removal of most nuclear and cytosolic proteins. This pathway regulates transcription directly through epigenetic ubiquitination and through poly-ubiquitination that can lead to the removal of DNA-associated proteins^5,6^. Furthermore, earlier work by our group and others indicates that the turnover of transcriptional regulators is site-selective and specific to some of the DNA regions to which these proteins are bound. Nuclear degradation by the ubiquitin–proteasome system is therefore not only target protein-selective, but also displays spatial specificity^7,8^.

The ubiquitin–proteasome system allows for specific ubiquitination of proteins through E3 ubiquitin ligases, of which there are around 600 subunits encoded in the human genome. However, these subunits often assemble into larger complexes with multiple variable subunits, increasing the actual number of functional E3 complexes multifold through combinatorial diversity. Cullin-RING ligase complexes represent about half of the encoded E3 genes, making it the largest family of ubiquitin ligases^9–13^.

Cullin ligase complexes are comprised of ubiquitin-conjugating enzymes, adapter proteins, substrate recognition factors, and the eponymous cullin proteins. Cullins are rigid, rod-like proteins that act as structural scaffolds^14^. Cullin ubiquitin ligases impact a variety of vital cellular functions, such as cell cycle progression, signaling, and DNA repair. Their specific role in transcriptional regulation is less well understood^13,15^.

Cullins have recently garnered therapeutic interest for their role in PROTAC- and IMiD-based protein removal^16^. Chemical linkers can be used to connect a cullin ligase complex with a specific substrate protein. In clinical practice, this approach is utilized to treat cancers. In particular, the complex consisting of CUL4 and the substrate receptor cereblon has shown promise by eliminating oncogenic transcription factors^17–19^. The general advantage of cullin-mediated protein removal with PROTACs is that it enables inhibition through degradation of previously undruggable polypeptides. With this increased attention on cullins, we hypothesized that cullin complexes might be involved in ubiquitination and perhaps removal of transcriptional regulators in a site-specific manner and at defined chromatin locations. In addition, recent studies described distinct ubiquitination patterns in the nucleus versus the cytosol, and we therefore sought to understand the spatial specificity of nuclear cullin ligase complexes^20,21^.

In this study, we investigate the intracellular distribution of the cullins CUL1, 2, 3, 4A, 4B, 5, and 7. We further analyze their chromatin-association, their likely interactions with other transcription factor networks, and the downstream genes that they regulate. Our results show that the spatial distribution of CUL4A and CUL4B is mutually exclusive. Further, we show strong chromatin-association of CUL1, especially at genes under control by the transcription factor c-MYC, and at promoters with high levels of protein turnover. CUL1 represses a subset of these genes that control mitochondria and RNA splicing, and CUL1-deficient cells display signs of mitochondrial stress.

## Results

### Expression of cullins

**Figure 1:**
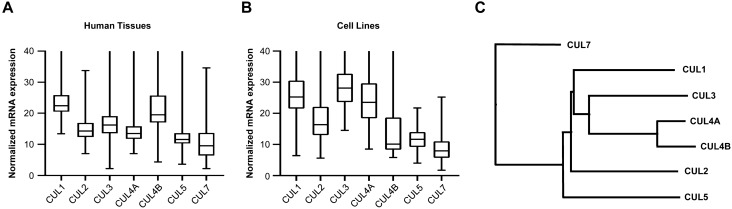
Cullin gene expression in human cells. (**A**) Box plot comparison of cullin expression in 62 human tissues shows highest expression of CUL1 and CUL4B. (**B**) CUL1 and CUL3 are the most highly expressed cullins across 64 human cell lines. The y-axis depicts consensus normalized mRNA expression levels (“NX”) based on The Human Protein Atlas (v.19.3). (**C**) Cullin paralogs have unique functions despite a high degree of amino acid sequence similarity. CUL7 is the most divergent with a combined amino acid sequence identity and similarity of 53.4% compared to CUL1.

To better assess the relative contribution of the ubiquitously expressed cullin backbone proteins to cellular function, we analyzed the individual cullin transcripts in 62 different human tissues from The Protein Atlas^22^. CUL1 and CUL4B are the two most highly transcribed cullin genes in primary human tissues (Fig. 1A). When comparing 64 human cell lines, CUL1 and CUL3 are the two most highly expressed cullins (Fig. 1B). Both results indicate that CUL1 is a major cullin in primary and tissue culture cells.

Despite the presence of seven paralogs, cullins still have unique functions. For instance, CUL1 has been well studied as part of the SCF complex (SKP1, CDC53/Cullin, and F-box proteins) that regulates cell cycle progression and signaling^23,24^. Notwithstanding the fact that CUL1 and its most divergent paralog CUL7 feature a combined 53.4% amino acid sequence similarity and identity (Fig. 1C), both are essential for viability and cannot be rescued by the presence of any other cullin^25–27^. One explanation is that these gene paralogs inhabit exclusive subcellular locations.

**Figure 2:**
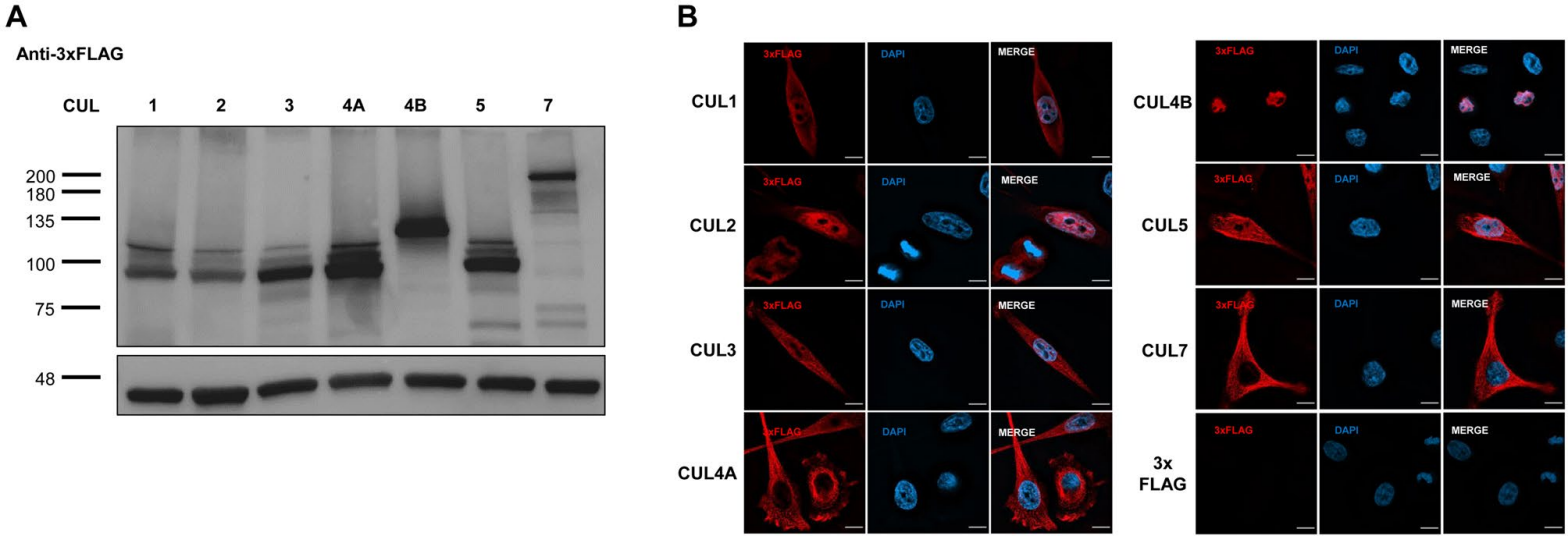
3xFLAG-Cullin expression and localization in HeLa cells. (**A**) Cullins were N-terminally tagged with 3xFLAG tag. HeLa cells were transfected individually with each 3xFLAG-Cullin, lysed, normalized by Bradford assay, and probed for 3xFLAG by immunoblot. Loading control is β-actin. Molecular weight is depicted in kDa. Uncropped immunoblots are shown in Suppl. Figure 1. (**B**) HeLa cells were transfected with each 3xFLAG-Cullin and immunofluorescence microscopy was performed against 3xFLAG at × 100 magnification. CUL1, 2, 3, and 5 are expressed in both the nucleus and cytoplasm, while CUL4A and 7 are largely excluded from the nucleus. CUL4B is exclusively nuclear. Size bar indicates 10 μm. Shown are representative images.

To determine the sites of individual cullin activities, we analyzed their intracellular expression patterns. It is noteworthy that there have been conflicting reports regarding for instance the cytoplasmic versus nuclear location of the closely related homologs CUL4A and CUL4B, respectively^17, 28–31^. These discrepancies are likely caused by the use of antibodies that cannot differentiate between all cullins, especially when they were raised against protein regions with a high degree of homology. To avoid cross-reactivity of primary antibodies, we individually tagged the N-termini of the seven cullins with a 3xFLAG domain. In previous structural and functional studies, N-terminal tags did not interfere with cullin biology^32–34^. The 3xFLAG domain allows for high fidelity isolation and visualization of tagged proteins using biochemical assays, microscopy, or chromatin-immunoprecipitation. We optimized DNA transfection with plasmids encoding the tagged constructs for similar expression levels of all seven cullins in human HeLa cells (Fig. 2A and Suppl. Figure 1) and identified their subcellular locations using immunofluorescence detection. CUL1, 2, 3, and 5 were expressed in the cytoplasm as well as the nucleus. CUL4A and CUL7 were predominantly excluded from the nucleus, while CUL4B was entirely nuclear (Fig. 2B).

### DNA association of cullins

**Figure 3:**
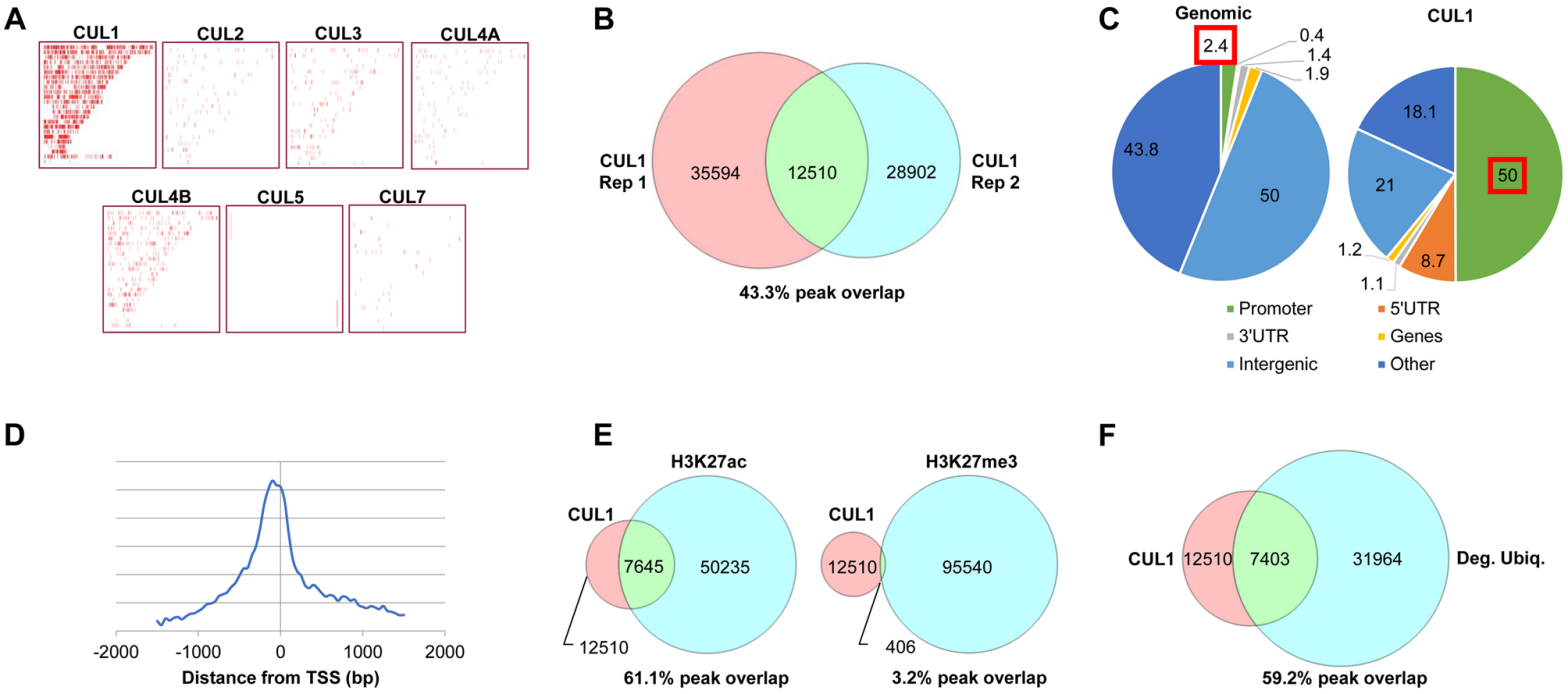
3xFLAG-Cullin Chromatin Immunoprecipitation-sequencing (ChIP-seq). (**A**) ChIP-seq of HeLa cells expressing each of the 3xFLAG-Cullins show that only CUL1 and CUL4B substantially associate with DNA. Marks indicate peaks with chromosomes listed from 1 to 22 and X, Y (top to bottom). The graph was generated with MACS2 and CEAS. (**B**) A comparison of CUL1 DNA binding peaks of two independent biological replicates. (**C**) CUL1 DNA affinity is enriched for promoter regions compared to the overall genomic prevalence of promoters (50% vs. 2.4%, *p* = 2.3E−322)^61^. Indicated are pie chart percentages comparing the entire genome (left) with CUL1-associated regions (right). Data was calculated with MACS2 and CEAS. (**D**) CUL1 peak distribution upstream of transcription start sites (TSS) shown as relative density plot. (**E**) CUL1-associated DNA regions are significantly enriched for H3K27ac, but are devoid of previously reported H3K27me3 marks (*p* < 2E−300, Chi-squared test with Yates’ correction). (**F**) CUL1 peaks are significantly enriched at sites of proteasome-dependent degradation. ChIP peaks from 3xFLAG-Ubiquitin-expressing HeLa cells treated with proteasome inhibitor represent degradation-prone ubiquitination^8^ “Deg. Ubiq.” (*p* < 2.33E−308, Chi-squared test with Yates’s correction).

To identify ubiquitin ligases that catalyze the ubiquitination and possibly degradation of DNA-associated transcription factors, we first assessed how the seven cullins might directly affect gene regulation through chromatin association. We used Chromatin Immunoprecipitation (ChIP) to determine the DNA affinity of all seven cullins in HeLa cells. Unsurprisingly, we found that cytoplasmic CUL4A and CUL7 did not interact with DNA. When testing cullins with exclusive or partial nuclear expression, only CUL1 and CUL4B substantially associated with DNA (Fig. 3A). In particular, CUL1 displayed reproducible, genome-wide peaks by ChIP-sequencing (ChIP-seq) (Fig. 3B). These findings are confirmed by earlier work that showed nuclear expression of CUL1 in human cell lines^32^ and chromatin association in *S. cerevisiae*^35^.

CUL1 peaks were significantly enriched at promoter regions upstream of transcription start sites (Fig. 3C,D). Cullins do not possess DNA binding domains and CUL1 is likely indirectly tethered to chromatin. Such indirect binding increases the functional space CUL1 controls. Further, CUL1 can bridge substrate proteins and ubiquitinating enzymes over distances of 100 Å^14,36^. On the compacted DNA solenoid, this distance translates to a linear DNA length of approximately 3,000 bp^37^. Thus, to identify potential DNA regions under control of CUL1, we extended the CUL1 peaks by 3 kb in either direction. These CUL1 regions strongly overlapped with the active chromatin mark H3K27ac and excluded the repressive mark H3K27me3 (Fig. 3E).

We previously defined DNA sites associated with high protein turnover by performing ChIP against ubiquitin. Our studies demonstrated that the addition of a proteasome inhibitor further increases the signal by enhancing degradation-prone ubiquitination in contrast to non-degradative ubiquitination^8^. Overlaying such a nuclear degradation map in HeLa cells with DNA regions under CUL1 control revealed a 59.17% overlap, suggesting that the majority of CUL1-associated sites are also sites of detectable protein degradation (Fig. 3F). Conversely, the CUL1-associated sites represent 23.16% of all genomic protein degradation sites in HeLa cells.

**Figure 4:**
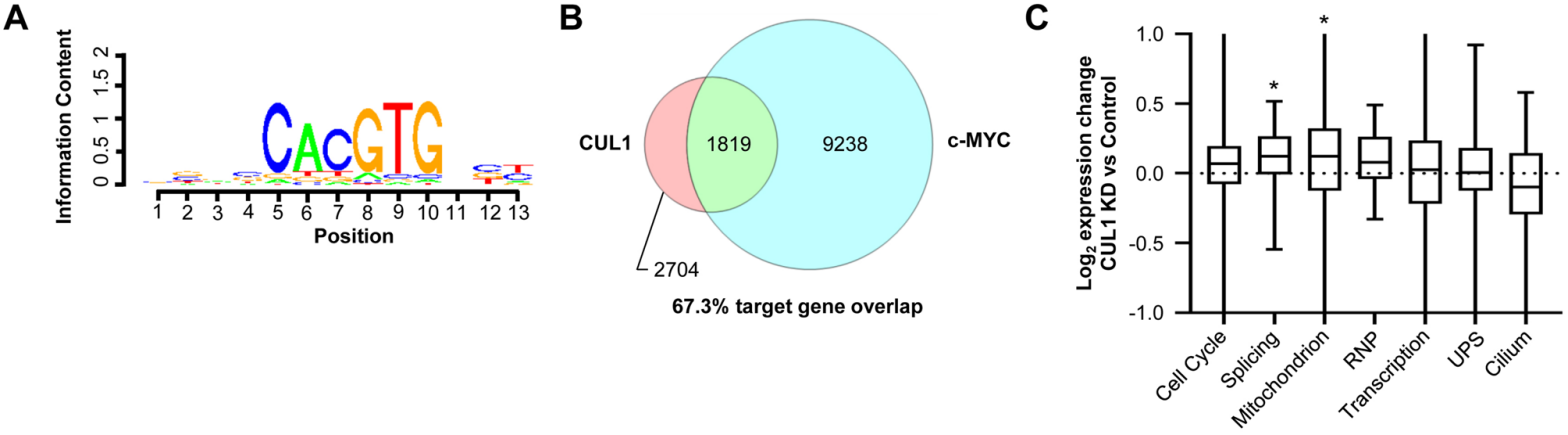
CUL1 target gene analysis. (**A**) CUL1 peaks are significantly enriched for the E-box DNA binding motif of the c-MYC/MAX heterodimer (*p* = 1.00714E−30)^69^. (**B**) Confirming the in silico motif enrichment, we also found significant overlap of CUL1 target genes with previously reported c-MYC target genes in HeLa cells (*p* < 2.06E−19, Chi-squared test with Yates’ correction). (**C**) Nuclear-encoded mitochondrial genes (*p* < 6.67E−7, Wilcoxon rank-sum test) and splicing-associated genes (*p* < 4.20E−6) are significantly upregulated in CUL1-deficient HeLa cells, as shown in this box plot. RNP refers to ribonucleoprotein complex genes; UPS refers to ubiquitin–proteasome system genes. Asterisks denote statistical significance. Gene ontologies were defined with DAVID^41^.

To identify the potential degradation targets or protein networks controlled by CUL1 activity, we examined CUL1-associated areas for known DNA binding motifs. We found the E-Box, a hallmark motif of the c-MYC/MAX heterodimer, to be highly enriched within CUL1-associated sites (Fig. 4A)^38^. Further, 67.3% of CUL1 target genes showed c-MYC occupancy at their promoters, suggesting that genes controlled by c-MYC may also be regulated by CUL1^39^ (Fig. 4B).

### Proximal gene regulation by CUL1

To functionally assess how CUL1 affects the expression of potential target genes, we performed unbiased RNA-sequencing in control HeLa cells or cells in which CUL1 was stably knocked down. Probable CUL1 target genes can be divided into seven main gene ontologies based on CUL1 affinity close to the transcription start site (< 1 kb): cell cycle genes (146 genes, *p* < 5.7E−15 for gene ontology enrichment), genes involved in RNA splicing (74 genes, *p* < 2.5E−14), nuclear-encoded mitochondrial genes (210 genes, *p* < 2.2E−13), ribonucleoprotein complexes (75 genes, *p* < 3.4E−10), transcriptional regulators (379 genes, *p* < 1.3E−6), genes of the ubiquitin–proteasome pathway (123 genes, *p* < 1.6E−6), and genes involved in cilium biology (37 genes, *p* < 5.5E−4)^40,41^. Of these seven gene ontologies with CUL1 affinity, two subsets were significantly altered in their expression upon knockdown of CUL1: nuclear-encoded mitochondrial genes and genes encoding splicing factors were upregulated in CUL1-deficient HeLa cells (Fig. 4C).

**Figure 5:**
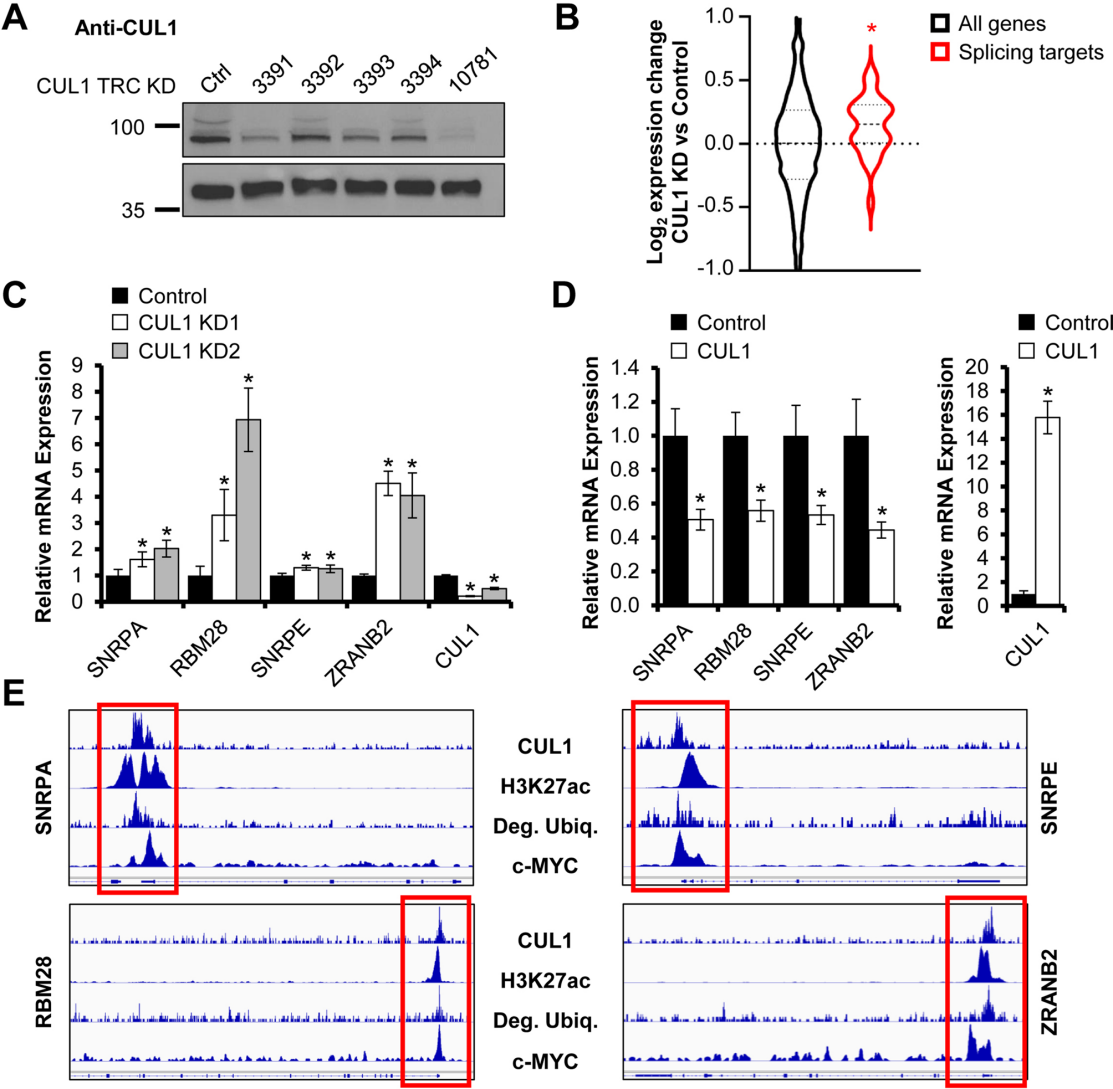
CUL1 represses splicing-associated genes. (**A**) HeLa cells stably transduced with shRNA against CUL1 show a stable reduction in CUL1 expression based on immunoblot against endogenous CUL1. Knockdown construct KD 1 represents TRCN0000010781; CUL1 protein levels are reduced to 11.82% relative to GAPDH control. Knockdown construct KD 2 is TRCN0000003391; CUL1 protein levels are reduced to 26.17% relative to GAPDH control. The left lane contains lysate from cells transduced with control vector TRCN0000241922. Protein lysates were normalized by Bradford assay. Densitometry was performed with ImageJ. Uncropped immunoblots are shown in Suppl. Figure 2. (**B**) Analysis of transcript expression changes upon CUL1 knockdown for genes that show *bona fide* peaks for c-MYC and both CUL1 replicates in their promoter regions. Splicing-associated genes show a significant upregulation upon CUL1 depletion (*p* = 2.46E−2, Wilcoxon rank-sum test). (**C**) CUL1 knockdown cells show a significant increase in transcripts of genes encoding splicing factors by RT-qPCR compared to cells expressing the TRC control vector (data are expressed as mean ± standard deviation, all significant *p* values < 1.21E−2, two-sided homoscedastic *t* test). RPS14 was used as reference transcript for ΔΔCt quantification. (**D**) HeLa cells transiently overexpressing 3xFLAG-CUL1 show a significant reduction in splicing-associated gene transcripts compared to the cells expressing the 3xFLAG vector alone (data are expressed as mean ± standard deviation, all significant *p* values < 2.42E−8, two-sided homoscedastic *t* test). RPS14 was used as reference transcript for ΔΔCt quantification. (**E**) Genome browser tracks of CUL1, H3K27ac, degradative ubiquitin, and c-MYC at select splicing-associated CUL1 and c-MYC target genes. Tracks from 3xFLAG-Ubiquitin-expressing HeLa cells treated with proteasome inhibitor represent degradative ubiquitination sites^8^, “Deg. Ubiq.”. Red boxes indicate promoter regions. Asterisks denote statistical significance.

**Figure 6:**
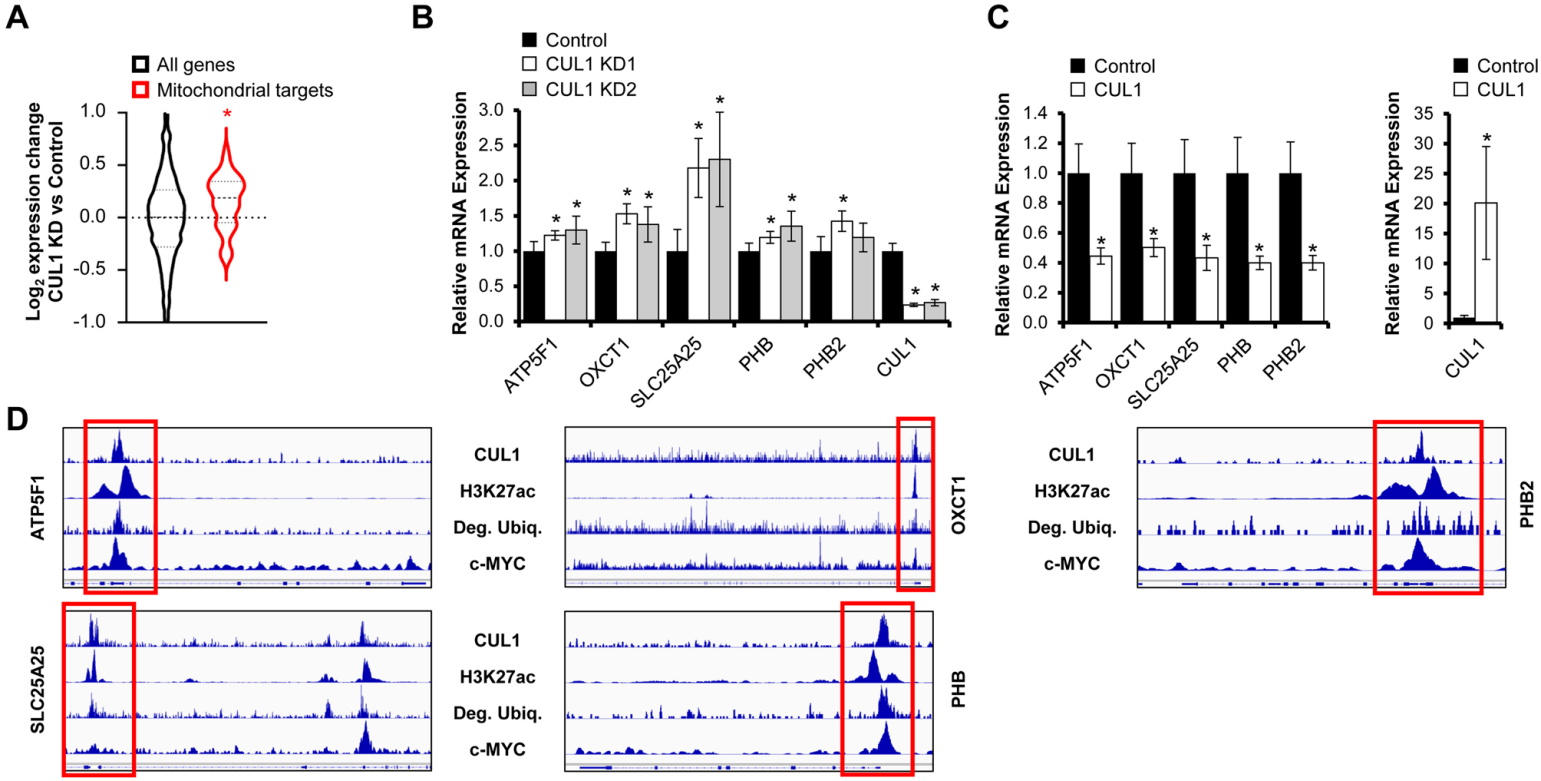
CUL1 represses mitochondrial genes. (**A**) Analysis of transcript expression changes upon CUL1 knockdown for genes that show *bona fide* peaks for c-MYC and both CUL1 replicates in their promoter regions. Mitochondrial genes show a significant upregulation upon CUL1 depletion (*p* = 5.58E−5, Wilcoxon rank-sum test). (**B**) CUL1 knockdown cells show a significant increase in nuclear-encoded mitochondrial transcripts by RT-qPCR compared to cells expressing the TRC control (data are expressed as mean ± standard deviation, all significant *p* values < 4.97E−2, two-sided homoscedastic *t* test). RPS14 was used as reference transcript for ΔΔCt quantification. (**C**) HeLa cells transiently overexpressing 3xFLAG-CUL1 show a significant reduction in nuclear-encoded mitochondrial gene transcripts compared to 3xFLAG vector-transfected cells (data are expressed as mean ± standard deviation, all significant *p* values < 1.50E−3, two-sided homoscedastic *t* test). RPS14 was used as reference transcript for ΔΔCt quantification. (D) Genome browser tracks of CUL1, H3K27ac, degradative ubiquitin, and c-MYC at select nuclear-encoded mitochondrial CUL1 and c-MYC target genes. Tracks from 3xFLAG-Ubiquitin-expressing HeLa cells treated with proteasome inhibitor represent degradative ubiquitination sites^8^, “Deg. Ubiq.”. Red boxes indicate promoter regions. Asterisks denote statistical significance.

CUL1 has a prominent role in cell cycle regulation. However, little is known about its function in transcriptional or metabolic control^42^. To validate whether CUL1 depletion alters expression of nuclear-encoded mitochondrial and splicing-associated genes, we used shRNA to generate two independent CUL1-deficient HeLa cell lines (Fig. 5A and Suppl. Figure 2). We then specifically analyzed genes for which we had established close affinity of CUL1 and c-MYC to the promoter regions by ChIP-seq. CUL1 knockdown cells displayed a significant increase in mRNA transcripts of these splicing-associated target genes and nuclear-encoded mitochondrial genes compared to cells expressing the control shRNA vector (Figs. 5B, 6A). Given that c-MYC-addicted cancer cells depend upon the spliceosome and that c-MYC drives mitochondrial biogenesis^43^, these data suggest an antagonistic relationship between c-MYC and CUL1. We performed RT-qPCR on select splicing-associated target genes and nuclear-encoded mitochondrial genes in dependence of CUL1 expression. Our studies confirmed the increased transcription of most target genes we tested in CUL1-deficient cells (Figs. 5C, 6B). Overexpression of CUL1 had the opposite effect and reduced expression of these target genes, suggesting the ubiquitin ligase has a repressor-like function on transcription from these c-MYC-associated gene promoters (Figs. 5D, 6C). Genome browser tracks show the close proximity of CUL1 affinity, c-MYC binding, and protein degradation at active (H3K27ac-positive) target promoters (Figs. 5E, 6D).

**Figure 7:**
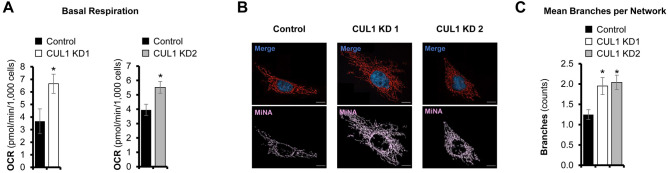
Mitochondrial phenotypes of CUL1-depleted cells. (**A**) CUL1 knockdown cells show higher levels of basal respiration compared to control cells. Oxygen Consumption Rate (OCR) is indicated as pmol/min/1,000 cells (data are expressed as mean ± standard deviation, all *p* values < 1.42E−2, two-sided homoscedastic *t* test). (**B**) CUL1 knockdown and control cells were treated with Mitotracker Red CMXRos and imaged at × 100 magnification. Mitochondrial network morphologies were analyzed by quantifying branching. Upper panel shows merged color channels; lower panel depicts mitochondrial network morphology as analyzed for branching. Size bar indicates 10 μM. (**C**) CUL1 knockdown cells show significantly more extensive branching, indicating mitochondrial fusion events (10 cells were analyzed per condition, data are expressed as mean ± SEM, all *p* values < 6.03E−3, two-sided homoscedastic *t* test). Shown are the mean numbers of branches per network as calculated with the MiNA tool^44^. Asterisks denote statistical significance.

To further investigate how CUL1-regulated transcription of metabolic genes affects cellular function, we analyzed the mitochondrial oxygen consumption in cells with normal or reduced CUL1 expression. Basal respiration was increased by an average of 60% in cells in which CUL1 was knocked down (Fig. 7A). In addition to increased respiration, we found evidence for elevated mitochondrial stress in the absence of CUL1. The morphology of mitochondrial networks showed significantly enhanced levels of fusion, which is consistent with damaged mitochondria that are attempting to repair and restore metabolic function^44,45^ (Fig. 7B,C). Overall, our results indicate that CUL1 is associated with the promoters of approximately 210 nuclear-encoded mitochondrial genes and a significant number of these genes are repressed by CUL1. De-repression increases mitochondrial activity, but also leads to morphological changes in mitochondria that are consistent with stress.

## Discussion

We here identify a novel role of the ubiquitin ligase CUL1 as a transcriptional repressor. A substantial number of genes controlled by c-MYC also show promoter association with CUL1. The promoters of these genes feature distinct ubiquitin peaks upon proteasome inhibition, indicating high levels of protein turnover. Our data suggest that CUL1 directly represses a subset of these genes involved in mitochondrial biology and splicing.

CUL1 and c-MYC both show synergistic function in cancers and can act as oncogenes^46,47^. While this seemingly contradicts the antagonistic function between CUL1 and c-MYC we describe here, a key role of CUL1 is, notably, to promote cell cycle progression. CUL1 contributes to this progression through bulk degradation of cell cycle regulators, a process fundamentally different from the DNA site-selective ubiquitination of proteins observed in our study. Further, c-MYC increases CUL1 expression and the repressive function of the ubiquitin ligase may act as a partial negative feedback to limit some c-MYC target genes^46^. The involvement of metabolic genes is of particular interest, given that the synchronization of mitochondrial biogenesis with cell cycle regulation is an emerging field. More research will be necessary to consolidate the synergistic and antagonistic roles of c-MYC and CUL1 and to parse out how CUL1’s transcriptional function correlates with the cell cycle status. The CUL1 knockdown cells in our study grew slightly slower than control cells, but showed no significant differences in cell cycle distribution (Suppl. Figure 3).

Ubiquitination occurs in various forms and does not necessarily lead to the degradation of a protein. Our study does not directly address whether CUL1 engages in degradative or non-degradative ubiquitination at promoter sites. However, we found evidence for protein degradation at the majority of CUL1 target DNA (Fig. 3F). Protein turnover was examined by quantifying the levels of DNA-associated ubiquitination upon proteasome inhibition^8^. Such treatment leads to a massive redistribution of ubiquitin, shuttling the limiting amounts of this protein from non-degradative to degradative use. Further evidence that CUL1 is specifically engaged in protein degradation can be found in numerous publications^10,46,48^. Interestingly, CUL1 has been described as a ubiquitin ligase that targets c-MYC for degradation through the substrate receptor FBXW7^49^. We have not found evidence of bulk changes in c-MYC protein levels after CUL1 knockdown or after the introduction of dominant-negative CUL1 (not shown). However, it is possible that c-MYC degradation by CUL1 occurs in a site-selective manner at specific promoters, in which case there may only be a negligible change to total c-MYC levels. We have previously observed such spatially selective degradation for other transcriptional regulators^8^. In summary, the identities of target proteins of chromatin-associated ubiquitination by CUL1 and their fates remain unsolved and are subjects of ongoing studies by our laboratory.

Previous reports on the subcellular locations of cullins were inconsistent, especially concerning the clinically relevant proteins CUL4A and CUL4B. Both cullins bind to cereblon^50^, a substrate-binding protein that triggers ubiquitination of the transcription factors IKZF1 and IKZF3 upon treatment with thalidomide or its derivatives. Degradation of IKZF1 and IKZF3 is therapeutically exploited in the treatment of hematological malignancies. This clinically relevant degradation through cereblon occurs in the nucleus^51^. Our results argue that this activity is mediated by CUL4B, not CUL4A. In support of our findings, an earlier report identified a nuclear localization sequence in CUL4B^29^. Except for CUL4A and CUL7, which are mostly excluded from the nucleus, all other cullins show some or specific expression in the nucleus. It is, therefore, possible that CUL2, 3, 4B, and 5 participate in the bulk ubiquitination of nuclear proteins and that CUL1 further engages in the site-selective ubiquitination of proteins at specific genomic regions.

Cullins represent the largest family of ubiquitin ligases. Here, we show a surprising variability in intracellular distribution of the seven cullins. Our data suggests that both CUL1 and CUL4B have the capacity to ubiquitinate DNA-bound proteins. In particular, CUL1 demonstrated the strongest association with chromatin and regulated the expression of genes that are under control of the transcription factor c-MYC. These results underscore that the specificity of ubiquitin ligases encompasses multiple dimensions: both the specificity for target proteins and the spatial specificity of where protein ubiquitination occurs are critical to the activity of the seven cullins. These features are of particular importance when it comes to targeting DNA-bound proteins, for which location dictates function.

## Methods

### Phylogram

Phylogram was created with Jalview^52^ based on Clustal-Omega protein sequence alignments^53^.

### Plasmids

Cullins were cloned into the 3xFLAG/pCMV7.1 vector (Sigma Aldrich, #E7533). CUL1 (GenInfo identifier #32307160), CUL3 (#380714661), CUL4B (#121114297), and CUL7 (#270265834) were cloned via NotI and KpnI sites. CUL2 (#311771638), CUL4A (#511772959), and CUL5 (#67514034) were cloned via SalI and XbaI. Plasmids are available through Addgene (#155019-155025).

### Cell culture

HeLa cells (ATCC, #CCL2) were cultured in DMEM, 1X (Dulbecco’s Modification of Eagle’s Medium with 4.5 g/L glucose and l-glutamine) (Corning, #10-017-CV) supplemented with 10% FBS Opti-Gold, performance enhanced (GenDEPOT, #F0900-050) and 1% penicillin streptomycin (Gibco, #15140-122).

### Western Blots

HeLa cells were transfected with 2 µg of 3xFLAG-CUL using Lipofectamine reagent 2000 (Invitrogen, #1168-019). Media was changed at 24 h, and at 48 h cells were washed with cold 1xPBS and frozen at − 80 °C. Cells were then thawed and lysed in RIPA cell lysis buffer (1X) with EDTA (Gendepot, #R4100-010) and protease inhibitor cocktail (1%, GenDepot, #P3100-010) for 1 h on ice with vortexing every 15 min. Lysates were pelleted at 4 °C at 14,000×*g* for 30 min and supernatant was collected in a separate tube. Protein concentration was determined using protein assay dye reagent concentrate (Bio-Rad, #500-00006) and normalized against bovine serum albumin (BSA) (Sigma-Aldrich, #A7906-50G). Protein was then mixed with 4 × Laemmli sample buffer (Bio-Rad, #161-0747) and β-mercaptoethanol (Sigma-Aldrich, #M6250-100ML) according to manufacturer specifications and loaded onto Mini-PROTEAN TGX stain-free gels (Bio-Rad, #4568123). Proteins were separated using the Bio-Rad PowerPac and 1X Tris/glycine/SDS buffer (Bio-Rad, #1610732) and subsequently transferred to Trans-Blot Turbo transfer pack membranes (Bio-Rad, #1704156) using Trans-Blot Turbo transfer system (Bio-Rad). Western blot analysis against 3xFLAG was carried out using monoclonal anti-FLAG M2 antibody produced in mouse (Sigma-Aldrich, #F1804-1MG) with β-actin rabbit monoclonal antibody (Cell Signaling Technology, clone D6A8) as a loading control. Western blot against CUL1 was performed with recombinant anti-Cullin1/CUL-1 antibody (Abcam, ab75817). The membrane was stripped in 62.5 mM Tris/10%SDS/0.5% β-mercaptoethanol at 37 °C for 30 min, re-equilibrated in 5% milk/TBST and re-probed as above with anti-GAPDH antibody—loading control (HRP) (Abcam, ab204481). SuperSignal West Pico PLUS chemiluminescent substrate (Thermo Scientific, #34577) was used to detect horseradish peroxidase (HRP)-conjugated proteins, and bands were visualized using the Bio-Rad ChemiDoc imaging system.

### Lentiviral production and CUL1 knockdown

TRC Lentiviral shRNA vectors were acquired from Horizon Discovery (Cat# RHS4533-EG8454; TRCN0000003391 and TRCN0000010781), using TRCN0000241922 as negative control vector. Other tested vectors include TRCN0000003392, TRCN0000003393, and TRCN0000003394. COS-1 cells (ATCC, #CRL-1650) were transfected with Lipofectamine reagent 2000 (Invitrogen, Cat #1168-019), 2 µg TRC shRNA vector and packaging plasmids. Virus was concentrated with Lenti-X Concentrator (Takara Bio, #631231) and treated with Polybrene (2 µg/mL, Millipore, #TR-1003-G). HeLa cells were infected with virus and subjected to puromycin selection (2 µg/mL, Gibco, #A11138-03).

### Immunofluorescence microscopy

Coverslips were autoclaved then treated with 0.1 µg/mL poly-d-lysine (Millipore, #A-003-E). HeLa cells were plated onto the coverslips, then transfected as described. Cells were fixed with 4% para-formaldehyde in PBS (Thermo Scientific, #28906) for 15 min, permeabilized with 0.5% Triton X-100 (Fisher Scientific, #9002-93-1) for 15 min, and blocked with 10% BSA (Sigma-Aldrich, #A7906-50G) for 1 h. Cells were then incubated with monoclonal anti-FLAG M2 antibody produced in mouse (see above) in 3% BSA in PBS for 1 h and AlexaFluor 594 goat anti-mouse IgG (H + L) (Invitrogen, #A11005) in 3% BSA in PBS for 45 min at 37 °C. Coverslips were mounted onto slides with ProLong Gold antifade reagent with DAPI (Invitrogen, #P36935) and sealed with clear nail polish. Images were taken in z-stacks at 100 × using the Zeiss CellDiscoverer7 and processed with the pre-installed Zeiss ZEN 3.1 (blue edition) software (https://www.zeiss.com/microscopy/us/products/microscope-software/zen.html). Settings: “Deconvolution (Defaults—Excellent)”.

### Chromatin immunoprecipitation (ChIP)

ChIP experiments with the 3xFLAG tag were performed as previously published^8^. In short, HeLa cells were grown in T175 flasks and harvested at 90% confluency. Each flask contained approximately 5 million cells and at least 10 million cells were harvested for each experimental condition. 3F-Ubiquitin ChIP was performed with stably transduced HeLa cells^8^. 3F-Cullin ChIP was performed with HeLa cells that were transfected with Lipofectamine 2000 (Thermo Fisher, #11668019) 48 h prior to harvest with 25 µg of 3F-Cullin and 5 µg of a GFP spike-in control vector to validate consistent transfection efficiency across different cullin constructs at > 80%. For 3F-Ubiquitin ChIP, proteasome inhibition was performed for 3 h prior to ChIP with 25 µM lactacystin or 0.1% v/v DMSO control (Cayman Chemical, #70980). Cells were washed and fixed in 1% para-formaldehyde in PBS (Thermo Scientific, #28906) at room temperature for 10 min, followed by quenching with glycine. Cells were manually detached by scraping and washed prior to lysis. 5 million cells were lysed per 5 mL dilution buffer (150 mM NaCl, 20 mM Tris pH 7.4, 2 mM EDTA) with the addition of Triton X-100 (1%, VWR, #IB07100), protease inhibitor cocktail (1%, Gendepot, #P3100-010), and RNase cocktail (1%, Thermo Fisher, #AM2288) for 10 min at 4 °C with constant mixing. Nuclei were isolated through centrifugation (350×*g*, 5 min, 4 °C) and immediately sonicated in dilution buffer containing 0.04% SDS, RNase, and protease inhibitor cocktail using a Bioruptor Pico water bath sonicator (Diagenode) at 4 °C. Shearing was optimized to yield DNA fragments of 200–500 bp. After removal of insoluble material through centrifugation, the nuclear lysate was aliquoted for input material or diluted to 0.01% SDS and immunoprecipitated over night with monoclonal anti-FLAG M2 antibody produced in mouse (see above) and protein G beads (Thermo Fisher, #10003D) that were blocked with DNA-free BSA (Thermo Fisher, #15561020). The following day, beads were washed twice with Tris-based buffer (see above) and eluted with 3xFLAG peptide for 15 min at room temperature (Sigma Aldrich, #F4799). Input and ChIP material was then de-crosslinked over night at 65 °C in the presence of 5% proteinase K (Thermo Fisher, #AM2546). Finally, DNA was recovered with Qiagen’s MinElute PCR kit (#28006). Size selection was performed prior to library preparation using AMPure XP beads (Beckman Coulter, #A63880).

### Next generation sequencing

The Genomic and RNA Profiling Core at Baylor College of Medicine performed next generation sequencing as previously described^54–57^. Libraries for ChIP-seq were synthesized and prepared for multiplexing according to New England BioLabs’ protocol for Illumina sequencing (Ultra Next DNA library prep kit I and II, #E7370S and #E7645S). As indexing primers, we used NEBNext Multiplex oligos (#E7335S and #E7500S). Libraries for RNA-seq were synthesized and prepared for sequencing with the KAPA stranded RNA-seq kit with RiboErase (HMR) (Roche, #KK8483) with ERCC ExFold RNA spike-in mixes (Thermo Fisher, #4456739). Indexing primers for RNA-seq were custom-synthesized by IDT.

ChIP-Seq: The Genomic and RNA Profiling Core first conducted sample quality checks using the NanoDrop spectrophotometer and Agilent Bioanalyzer 2100 (High Sensitivity DNA Chip, #5067-4626). To quantitate the adapter ligated library and confirm successful P5 and P7 adapter incorporations, we used the Applied Biosystems ViiA7 real-time PCR system and a KAPA Illumina/universal library quantification kit (#KK4824). We then sequenced the libraries on the Nextseq500 system using the high output v2.5 flowcell.

Library quantification by qPCR and Bioanalyzer: A qPCR assay was performed on the libraries to determine the concentration of adapter ligated fragments using the Applied Biosystems ViiA7 quantitative PCR instrument and a KAPA library quant kit (#KK4824). All samples were pooled equimolarly and re-quantitated by qPCR, and also re-assessed on the Bioanalyzer.

Cluster Generation by Bridge Amplification: Using the concentration from the ViiA7 qPCR machine above, 1.8 pM of equimolarly pooled library is loaded onto a NextSeq 500 high output v2.5 flowcell (Illumina #20024906) and amplified by bridge amplification using the Illumina NextSeq 500 sequencing instrument. PhiX Control v3 adapter-ligated library (Illumina, #FC-1103001) is spiked-in at 1% by weight to ensure balanced diversity and to monitor clustering and sequencing performance. A single-end 75 cycle run was used to sequence the flowcell on a NextSeq 500 sequencing system to achieve a minimum of 25 million reads per sample. Fastq file generation and data delivery was achieved using Illumina’s Basespace sequence hub.

RNA-seq: The Genomic and RNA Profiling Core first conducted sample quality checks using the NanoDrop spectrophotometer and Agilent Bioanalyzer 2100 (high sensitivity DNA Chip, #5067-4626). To quantitate the adapter ligated library and confirm successful P5 and P7 adapter incorporations, we used the Applied Biosystems ViiA7 real-time PCR system and a KAPA Illumina/universal library quantification kit (#KK4824). We then sequenced the libraries on the Nextseq500 system using the high output v2.5 flowcell.

Library quantification by qPCR and Bioanalyzer: A qPCR assay was performed on the libraries to determine the concentration of adapter ligated fragments using the Applied Biosystems ViiA7 quantitative PCR instrument and a KAPA library quant kit (#KK4824). All samples were pooled equimolarly and re-quantitated by qPCR, and also re-assessed on the Bioanalyzer.

Cluster Generation by Bridge Amplification: Using the concentration from the ViiA7 qPCR machine above, 1.8 pM of equimolarly pooled library is loaded onto a NextSeq 500 high output v2.5 flowcell (Illumina, #20024907) and amplified by bridge amplification using the Illumina NextSeq 500 sequencing instrument. PhiX Control v3 adapter-ligated library (Illumina, #FC-1103001) is spiked-in at 1% by weight to ensure balanced diversity and to monitor clustering and sequencing performance. A paired-end 75 cycle run was used to sequence the flowcell on a NextSeq 500 sequencing system to achieve a minimum of 50 million reads per sample. Fastq file generation and data delivery was achieved using Illumina’s Basespace sequence hub.

### Data processing

ChIP-seq fastq files were processed with Cutadapt ver. 1.12^58^ and mapped to the HG19 genome with Bowtie ver. 1.0^59^. Peak calling was performed with MACS2 ver. 2.1.0.20140616^60^ with a false discovery rate < 0.05. Peaks were compared to input DNA as well as ChIP DNA from cells transfected with the 3xFLAG/pCMV7.1 control vector (Sigma Aldrich, #E7533). Mapping to functional genomic sites and target genes was performed with CEAS ver. 1.0.2^61^. Gene ontologies were defined with DAVID (https://david.ncifcrf.gov) ver. 6.8^40^. Target site and peak overlaps were analyzed with Bedtools ver. 2.23.0^62^ and fold enrichment was calculated based on randomized peaks of equal number and size and intra-chromosomal permutation. Wig files were created from MACS2 output with Samtools ver. 0.1.19-96b5f2294a^63^. Wig and bigwig files were visualized using the Integrative Genomics Viewer (IGV) version 2.3 from the Broad Institute^64–66^. The following ENCODE data was utilized: c-MYC (ENCFF045UZK, ENCFF224GZD), H3K27ac (ENCFF388WMD), and H3K27me3 (ENCFF252BLX)^67,68^. The c-MYC reference file used in this study is based on common peaks between both entries. Similarly, Venn diagram comparisons for CUL1 binding are based on common peaks between two biological ChIP replicates. As outlined in the manuscript, domains under CUL1 control were estimated by extending peak regions 3,000 bp in both directions. The analysis of gene expression changes by RNA-seq in Figs. 5B and 6A was performed using the *bona fide* peaks (not extended regions) of both CUL1 replicates and c-MYC. ChIP bed files were subjected to motif analysis using the SeqPos module in Cistrome^69^. Parameters were defined as sequencing positions *p* < 0.05, peak size 600 bp, using fold enrichment.

RNA-seq fastq files were processed with Cutadapt ver. 1.12 and mapped to the HG19 genome with TopHat2/Bowtie2 ver. 2.1.0^70^. Gene expression changes were quantified with Cufflinks and Cuffdiff ver. 2.1.1^71^.

### RNA extraction

RNA was extracted from confluent HeLa cells with RNeasy kit with RNAse-free DNaseI treatment (Qiagen, #74134 and #79254).

### RT-qPCR

RNA was subjected to RT-qPCR using Invitrogen SuperScript III Platinum SYBR Green one-step qRT-PCR kit with ROX (Thermo Fisher, #11746-500) according to the manufacturer’s instruction.

Primer sequences:

- ATP5F1_F: GGTGTAACAGGACCCTATGTACT
- ATP5F1_R: GAAGGTCTCTGCGCTAATCAC
- OXCT1_F: TGGAGATGACGTAAGGGAACG
- OXCT1_R: GGAGAGGGATTCCTATGCCCA
- SLC25A25_F: TGACCATCGACTGGAACGAGT
- SLC25A25_R: ACATCAAAGATCGTGGAATGCTT
- PHB_F: TGTCATCTTTGACCGATTCCG
- PHB_R: CTGGCACATTACGTGGTCGAG
- PHB2_F: GTGCGCGAATCTGTGTTCAC
- PHB2_R: GATAATGGGGTACTGGAACCAAG
- SNRPA1_F: GGTGCTACGTTAGACCAGTTTG
- SNRPA1_R: GTCCCTCACCTATACGGCATATT
- RBM28_F: ATGTCCGCATTGTCTTGCATC
- RMB28_R: GGCCATCCAGTTTAAGCCCA
- SNRPE_F: TGCAGCCCATCAACCTCATC
- SNRPE_R: GCCTTCTATCCGCATATTCACTT
- ZRANB2_F: GTGGTCGGGAGAAAACAACTG
- ZRANB2_R: CCCAATTCACATTGCTGCAAGT
- CUL1_F: AGCCATTGAAAAGTGTGGAGAA
- CUL1_R: GCGTCATTGTTGAATGCAGACA
- RPS14_F: CCATGTCACTGATCTTTCTGGC
- RPS14_R: TCATCTCGGTCTGCCTTTACC

### Oxygen consumption assays

Seahorse XFp cell culture miniplates (Agilent, #103025-100) were treated with Cell-Tak cell and tissue adhesive (0.024 mg/mL Corning, #354240) according to manufacturer specifications and 30,000 cells/well were plated. Agilent Seahorse XF base medium (#103193-100) was supplemented with 25 mM glucose, 2 mM sodium pyruvate, and 2 mM l-glutamine. Basal respiration was normalized by cell count.

### Mitotracker and MiNA analysis

HeLa cells were incubated with 500 nM Mitotracker Red CMXRos (Invitrogen, #M7512) for 30 min, then placed on coverslips, permeabilized, and mounted as described. Images were taken as z-stacks at 100 × with the Zeiss CellDiscoverer7 and processed with Zeiss ZEN 3.1 (blue edition) Deconvolution (Defaults—Excellent)”. Using ImageJ, images were converted to RGB, auto-thresholding was applied (yen algorithm), and pictures were subjected to MiNA analysis^44^.

### Cell cycle analysis

HeLa Control or CUL1 knockdown cells were transiently transfected for 48 h with Lipofectamine 2000 (Thermo Fisher, #11668027) and the FastFUCCI construct^72^ on 6-well plates. Microscopy was performed on a live-cell imager (CD7, Zeiss) at 20 × magnification. Analysis of 6 × 6 fields of view per cell type was performed with Zeiss ZEN 3.1 software. Cells were grown in phenol red-free DMEM medium with supplementation. The plasmid pBOB-EF1-FastFUCCI-Puro was a gift from Kevin Brindle & Duncan Jodrell (Addgene plasmid # 86849; https://n2t.net/addgene:86849; RRID:Addgene_86849).

## Supplementary information

Supplementary Information 1.

Supplementary Information 2.

## Data Availability

Raw and processed ChIP-seq files are available at the Gene Expression Omnibus under GSE147426.
