# Supplementary material for: The ubiquitin ligase Cullin-1 associates with chromatin and regulates transcription of specific c-MYC target genes
Source: Sci Rep. 2020 Aug 18;10:13942. doi: 10.1038/s41598-020-70610-0 (PMC7435197; doi:10.1038/s41598-020-70610-0)
Supplement: Supplementary file 2 — Supplementary Information 2. [file 41598_2020_70610_MOESM2_ESM.pdf]

Supplementary Figures

The Ubiquitin Ligase Cullin-1 Associates with Chromatin and Regulates Transcription of Specific c-MYC Target Genes

Melanie A. Sweeney[1,2,3,4,5], Polina Iakova[2,3,4,5], Laure Maneix[2,3,4,5], Fu-Yuan Shih[2,3,4,5], Hannah E. Cho[2,6], Ergun Sahin[2], Andre Catic[1,2,3,4,5,7,*]

1. Department of Molecular and Cellular Biology, Baylor College of Medicine, Houston, TX, USA
2. Huffington Center on Aging, Baylor College of Medicine, Houston, TX, USA
3. Stem Cells and Regenerative Medicine Center, Baylor College of Medicine, Houston, TX, USA
4. Dan L. Duncan Cancer Center, Baylor College of Medicine, Houston, TX, USA
5. Center for Cell and Gene Therapy, Baylor College of Medicine, Houston, TX, USA
6. Rice University Undergraduate School of Social Sciences, Houston, TX, USA
7. Michael E. DeBakey Veterans Affairs Medical Center, Houston, TX, USA
*Correspondence and requests for materials should be addressed to A.C. (andre.catic@bcm.edu)

Suppl. Fig. 1

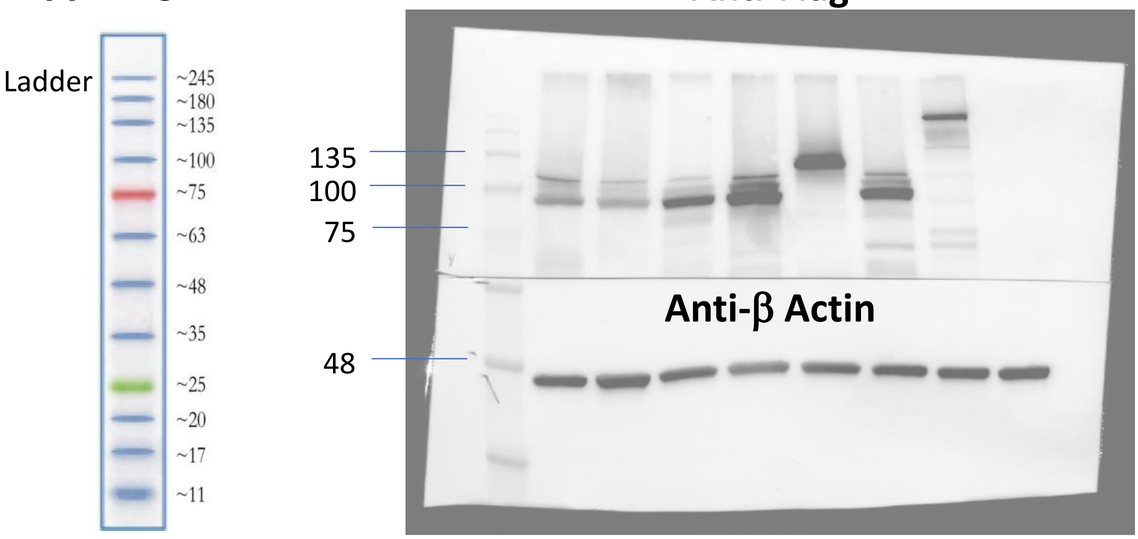

Suppl. Fig. 2

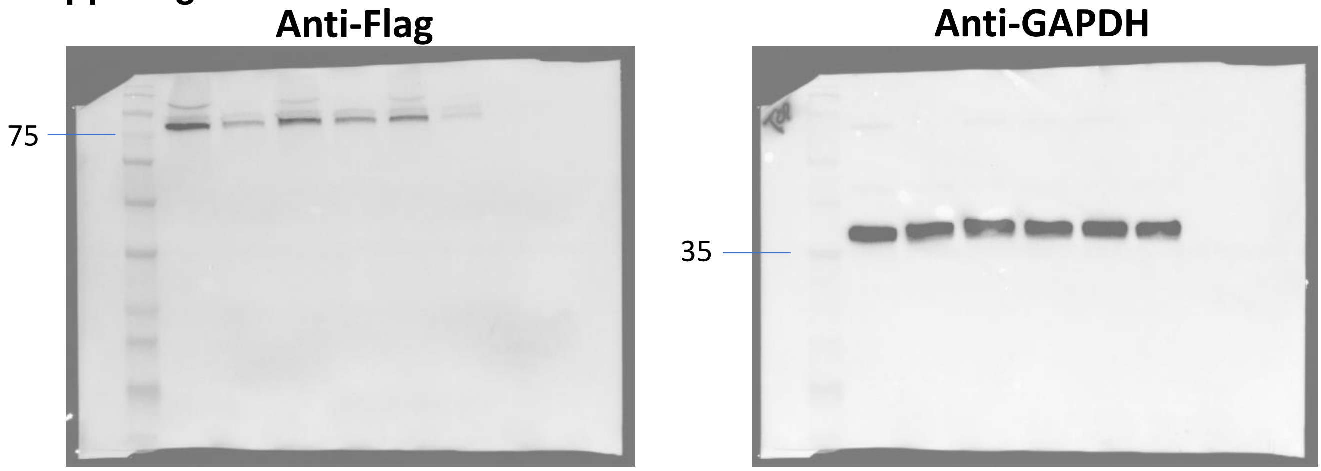

Supplementary Figures 1 & 2: Original pictures of immunoblot membranes. The cropped images were shown in Figure 2A (Suppl. Fig. 1) and Figure 5A (Suppl. Fig. 2). The molecular weight reference is shown on the top left in kDa. This marker was used in the leftmost lane of each blot.

Suppl. Fig. 3

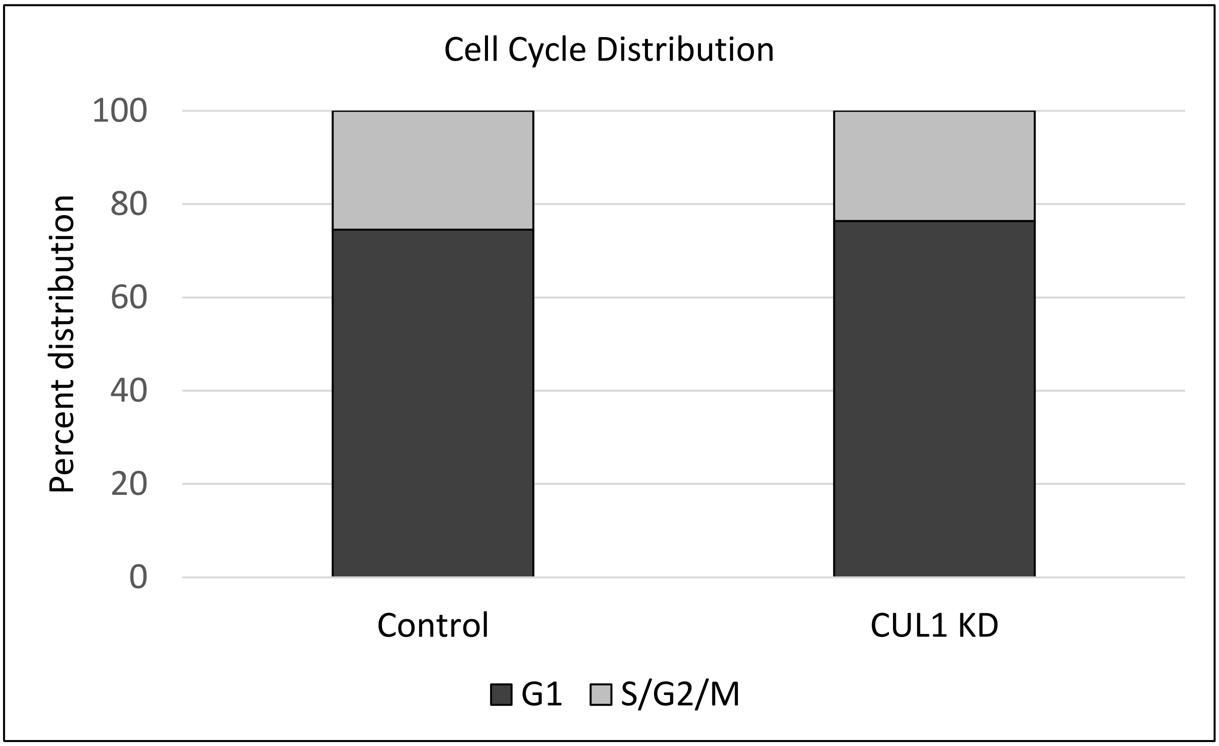

Supplementary Figure 3: Cell cycle analysis of HeLa Control and CUL1 knockdown cells, transiently transfected with the FastFUCCI construct. Over 200 cells were scored for each cell type, and cell cycle states were assessed based on expression of the Kusabira Orange 2 reporter for the G1 phase and Azami Green for the S/G2/M phases. CUL1 KD cells grew slightly slower than Control cells, but overall cell cycle phase distribution appeared similar in both cell types.
